# Agarose/Spherical Activated Carbon Composite Gels for Recyclable and Shape-Configurable Electrodes

**DOI:** 10.3390/polym11050875

**Published:** 2019-05-14

**Authors:** Jong Sik Kim, Ju-Hyung Kim, Younghyun Cho, Tae Soup Shim

**Affiliations:** 1 Department of Energy Systems Research, Ajou University, Suwon 16499, Korea; kjsik@ajou.ac.kr; 2 Department of Chemical Engineering, Ajou University, Suwon 16499, Korea; 3 Department of Energy Systems, Soonchunhyang University, Asan 31583, Korea

**Keywords:** agarose, hydrogels, activated carbon, soft electrodes, conducting polymers

## Abstract

Soft electrodes have been known as a key component in the engineering of flexible, wearable, and implantable energy-saving or powering devices. As environmental issues are emerging, the increase of electronic wastes due to the short replacement cycle of electronic products has become problematic. To address this issue, development of eco-friendly and recyclable materials is important, but has not yet been fully investigated. In this study, we demonstrated hydrogel-based electrode materials composed of agarose and spherical activated carbon (agar/SAC) that are easy to shape and recycle. Versatile engineering processes were applied thanks to the reversible gelation of the agarose matrix which enables the design of soft electrodes into various shapes such as thin films with structural hierarchy, microfibers, and even three-dimensional structures. The reversible sol–gel transition characteristics of the agar matrix enables the retrieval of materials and subsequent re-configuration into different shapes and structures. The electrical properties of the agar/SAC composite gels were controlled by gel compositions and ionic strength in the gel matrix. Finally, the composite gel was cut and re-contacted, forming conformal contact to show immediate restoration of the conductivity.

## 1. Introduction

Energy and environmental issues are global concerns. The development of energy-efficient eco-friendly electronic devices has been pursued by a broad array of researchers. A new electronic device form factor has emerged to meet industry needs for flexible, wearable, implantable, and 3D-printable electronic devices. There is a need for materials that can substitute traditional metal- and silicon-based materials. The short replacement cycle of electronic products has increased electronic wastes, which are problematic for the environment. In an effort to address the aforementioned issues, conductive polymers or composites have been investigated for use as flexible, healable, non-toxic, and biocompatible electrode materials [1,2,3,4]. Recyclable and shape-configurable electrode materials are in demand for use in future electronic devices that are designed to be disposable, such as single-use medical diagnostic devices. The development of conducting materials from biologically inert and soft polymer matrices with good electron transfer capabilities offers a promising strategy for recyclable and shape-configurable electrodes.

Polymer-based conducting materials have been developed previously either by combining conducting materials with a polymeric matrix or using conducting polymers alone. Several conducting materials have been tested thus far: (1) carbon-based materials, such as activated carbon, graphene, and carbon nanotubes; (2) conducting polymers, such as polypyrrole [5], polyaniline [6], and polythiophene [7]; and (3) metal oxides, such as RuO_2_ [8] and MnO_2_ [9]. Each material has its strengths and weaknesses with respect to durability, biocompatibility, and material cost. As a soft polymer matrix, hydrogels are promising candidates that satisfy requirements for flexibility, reusability, biocompatibility, and cost [10]. In addition, hydrogels absorb large amounts of water through sparse polymeric networks, which is advantageous for transferring ions and developing electronic devices that are integrated into the human body.

Agarose is a polysaccharide, biodegradable hydrogel extracted from seaweed. Due to its excellent biocompatibility, good mechanical strength, large pore size, and water absorption characteristics, it has been used as a matrix for transferring ions, nutrients, polymers, and proteins in various applications, such as cell culturing, gel electrophoresis, and protein purification. Recently, agarose has been used in electronic materials by combining it with carbon-based materials, such as graphene oxide [11], carbon nanotubes [12,13], or conducting polymers, such as polypyrrole [14,15], poly(3,4-ethylenedioxythiophene) (PEDOT) [16], or polyaniline [17,18]. A series of agarose-based electronic materials exhibited good conductivity around O(10^−4^)–O(10) S/cm as a polymer electrode. Agarose has also been used to develop flexible, solid electrolytes due to its excellent ion transfer capabilities [19]. Previous studies only focused on the material performances and not the ease of processability or recyclability, which is crucial for utilizing hydrogel-based conducting materials in practical uses.

In this paper, we prepared agarose/spherical activated carbon (agar/SAC) composite gels for use in recyclable and shape-configurable electrodes. As a conducting material, SAC was chosen for its high surface area per unit mass compared with other carbon-based materials. Brunauer–Emmett–Teller (BET) analysis of the SAC sample revealed that the surface area of the SAC was as high as 3314.59 m^2^/g, as shown in Figure S1. To fully utilize the shape-configurable nature of the agar matrix, a geometrically isotropic SAC sample was selected rather than a flake-type activated carbon. The spherical shape of the conducting material offered several advantages. The surface area of the SAC could be fully utilized because the individual SAC particles formed only limited contacts with the neighboring SAC particles, whereas the 2D flake-type carbon material formed stacks that significantly decreased their effective surface area. Material processing procedures, such as polymer/precursor extrusion and spin-casting, apply shear stresses that lead to 2D material stacking in specific directions causing the material to have anisotropic electrical properties. The combination of agar and SAC enabled preparation of conducting gels that could be formed into various shapes with consistent electrical properties. The electrical properties of the agar/SAC composite gels could be controlled by varying the composition of the SAC and the ionic strength of the aqueous solution. By using the reversible gelling characteristics, we demonstrated a series of recycling processes using a variety of engineering processes. Finally, easy recovery of electrical properties after mechanical damage due to conformal contact of materials was demonstrated.

## 2. Materials and Methods

Agarose powder (Samchun Pure Chemical Co., Ltd., Seoul, Korea), poly(dimethylsiloxane) (PDMS, Sylgard 184), sodium phosphate monobasic (NaH_2_PO_4_, Daejung Chemical & Metal Co., Ltd., Siheung, Korea), and sodium phosphate dibasic (Na_2_HPO_4_·12H_2_O, Daejung Chemical & Metal Co., Ltd., Siheung, Korea) were used as received. Spherical activated carbon (6 μm in diameter, SAC) was purchased from Asahi Organic Chemical Industry (Nagoya, Japan) and was filtered using a sieve with an aperture size of 150 μm to remove impurities.

The agar/SAC mixture was prepared by mixing 1 g agar powder with 9 g deionized (DI) water. Depending on the weight of DI water, 3, 5, 7, or 10 wt %, SAC was added to the agar solution. After vigorous mixing, the solution was heated in a water bath at 90 °C for 30 min. Finally, the mixture was stored at room temperature as a gel and was heated to 90 °C in a water bath prior to use. The agar/SAC mixture with a higher ionic strength was prepared using a phosphate buffer saline (PBS, pH 7) solution prepared by adding 0.138 g sodium phosphate monobasic and 0.303 g sodium phosphate dibasic to 200 mL DI water. Subsequently, 1 g agar powder was mixed with PBS rather than DI water.

Thermogravimetric analysis (TGA) was performed to analyze the composition of the agar/SAC composite gel using an instrument (SDT 2960, TA Instrument, New Castle, DE, USA). TGA was conducted by heating the sample from room temperature to 600 °C at a heating rate of 10 °C /min under nitrogen flow.

The electrical properties of the cubic agar/SAC composite gel were measured by preparing a 1 cm^3^ cubic polydimethylsiloxane (PDMS) mold using conventional soft lithography. Prior to agar/SAC molding, copper tape was attached to the sidewall of the cubic PDMS mold, the molten agar/SAC mixture was poured, and gelation proceeded at room temperature. Current–voltage (I–V) measurements were performed using a voltage sweep from 0.0 to 1.0 V with 0.01 V/point using a source measure unit (Keithley 2636B, Tektronix, Beaverton, OR, USA).

For image analysis of agar/SAC gel morphology and micropatterned structures, scanning electron microscopy (SEM, S4300, Hitachi, Tokyo, Japan) and optical microscopy (BX 43, Olympus, Tokyo, Japan) were used.

## 3. Results

### 3.1. Preparation of the Homogeneous Agar/SAC Mixture

**Figure 1:**
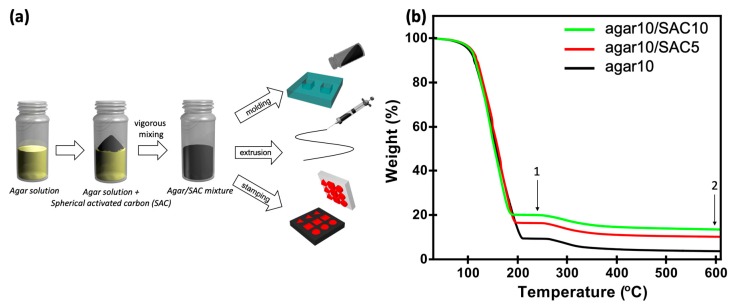
(**a**) Schematic diagram showing the preparation of the shape-configurable agarose and spherical activated carbon (agar/SAC) composite gels through various processes. (**b**) Thermogravimetric analysis (TGA) of the 10 wt % agarose gel as a control (agar10, black), 10 wt % agarose containing 5 wt % spherical activated carbon (agar10/SAC5, red), and 10 wt % agarose containing 10 wt % SAC (agar10/SAC10, green). Gels were fully dried near 230 °C (point 1), and agar was fully decomposed near 600 °C (point 2).

Agar/SAC composite gels can be prepared from a homogeneous agar/SAC mixture using various processes, as illustrated in Figure 1a. We prepared an agar/SAC mixture by mechanically mixing the agar solution at 90 °C with the SAC powder. The hydrophobic SAC powder was dispersed in the aqueous agar solution using a planetary centrifugal mixer (ARE-310, Thinky, Tokyo, Japan). Thermogravimetric analysis (TGA) was applied to the composite gels prepared with different compositions of agar and SAC, as shown in Figure 1b. All TGA data showed two gradual decreases in weight from 0–200 to 250–400 °C. The first phase was attributed to water evaporation, and the second phase was attributed to agar decomposition [20,21]. The weight at 230 °C was assumed to be the weight of the fully dried sample. We compared the weight differences between the fully dried samples and the sample obtained after complete decomposition of agar to verify the composition of agar and SAC in the composite gels. The weight of SAC in the composite gels, formulated to be 5 and 10 wt % SAC, were found to be 6.17% and 9.27% SAC, respectively, which closely corresponded to the composition of the original formulations, Table 1.

### 3.2. Reversible Shape Configuration of Agar/SAC Composite Gels using Various Engineering Processes

**Figure 2:**
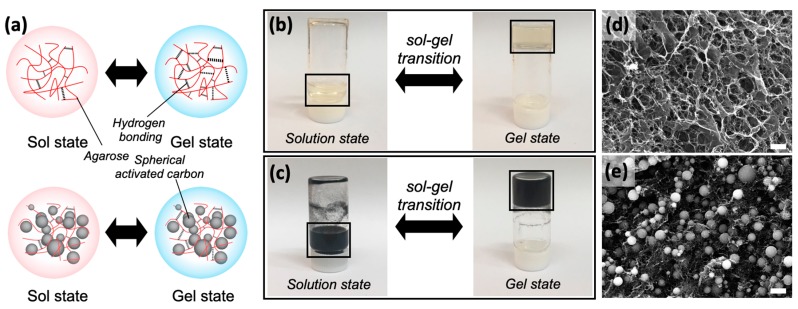
(**a**) Schematic illustration of the thermal sol–gel transition of the pure agarose and the composite gel, having fine and sparse agarose networks due to abundant and deficient hydrogen bonding, respectively. (**b**) Photos of the agarose sol at 90 °C (left) and the gel at room temperature (right), (**c**) Photos of the agar/SAC mixture sol at 90 °C (left) and the composite gel at room temperature (right). (**d**,**e**) Scanning electron microscopy images of cross-sectional views of freeze-dried (**d**) the agarose gel and (**e**) the agar10/SAC10 composite gel. Scale bars in (**d**,**e**) indicate 1 and 10 μm, respectively.

The agar/SAC composite gels underwent a reversible sol–gel transition upon heating/cooling, which was crucial for developing shape-configurable electrode materials. Agar could be physically crosslinked below the melting temperature as repeating units were bonded through hydrogen bonds [22], as illustrated in Figure 2a. The agar solution and agar/SAC mixture showed the same reversible sol–gel transition behaviors at 90 °C, Figure 2b,c, with slightly different mechanical strengths. Whereas the pure agar gel formed a fine polymer network, the agar/SAC composite gels formed rather sparse networks, as shown in Figure 2d,e. The network differences were attributed to the high volume-to-weight ratio of the SAC microspheres, which occupied a significant volume of the composite gels and interrupted the hydrogen bonds among the agar polymers.

**Figure 3:**
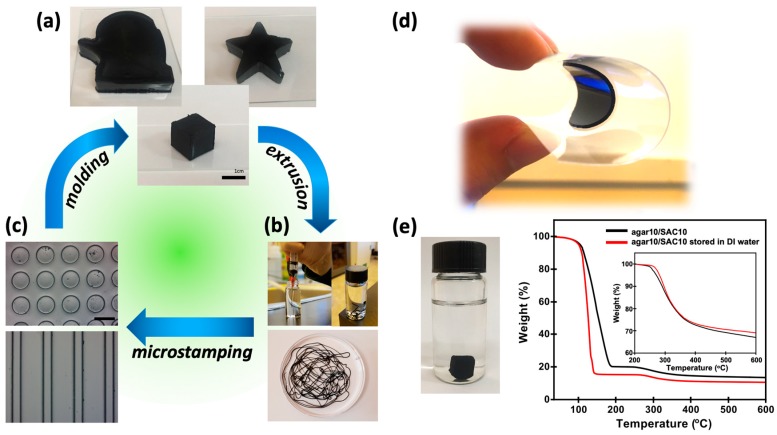
(**a**–**c**) Image gallery of the recyclable and shape-configurable agar/SAC composite gels using various engineering processes. (**a**) Photos of the agar10/SAC10 composite gels with a mitten (left), star (right), or cube (middle) shape, prepared using the molding process. The scale bar indicates 1 cm. (**b**) Photos of the fibrous agar10/SAC10 composite gels stored in water and prepared using an extrusion process. (**c**) Optical microscopy images of the agar10/SAC10 composite gel prepared in circle (top) or line (bottom) patterns prepared using a microstamping process. The scale bar indicates 500 μm. (**d**) Photo of the agar/SAC composite gel prepared on a flexible polyethylene terephthalate (PET) substrate. (**e**) TGA analysis of the as-prepared (black) and water-stored (red, left image) agar10/SAC10 composite gels. (inset) The weight of the fully dried agar10/SAC10 composite gel (W_dry_) compared to the normalized weights of the two samples.

The structural integrity of the agar/SAC composite gel is of great interest for designing shape-configurable electrodes. Thanks to its reversible and quick gelation characteristics, agar/SAC composite gels can be prepared and recycled into various shapes using different engineering processes. We demonstrated reconfiguration of agar/SAC composite gels by repetitive gelation and melting processes, as shown in Figure 3. The shape of the agar/SAC composite gel was configurable by molding the agar/SAC mixture and allowing gelation at room temperature. We formed cubic, star-shaped, and mitten-shaped agar/SAC composite gels to demonstrate the shape configurability, as shown in Figure 3a. The quick gelation characteristics also enabled the continuous production of fibrous composite gels by extrusion of the agar/SAC mixture through a syringe. As the molten agar/SAC mixture at 90 °C was injected into the water at room temperature, gelation took placed within a few seconds, resulting in fibrous agar/SAC composite gels, Figure 3b. The dimensions and mechanical properties of the fibrous agar/SAC composite gels were controlled simply by the inner diameter of the syringe needle and the agar composition, respectively, which enabled the production of textiles or 3D-printed electrodes. Hierarchically designed gel electrodes were prepared using microstamping. Line and dot array micromolds composed of poly(ethylene glycol) diacrylate were stamped using the agar/SAC composite gels, Figure 3c. Here, the hydrophilic master mold was selected to avoid the condensation of vapors generated from the hot molten agar/SAC mixture onto the master (see the Supporting Information Figure S2 for details). Micropatterning is potentially important for the design of gel-type electrodes with a structural hierarchy. Specifically, micron-scale patterned composite gels could provide synergetic effects with the porous SAC, thereby improving the contact area between the electrode and the electrolyte and increasing the efficiency of electron transfer in an energy device [23,24]. Finally, we prepared a film-type agar/SAC composite gel on a flexible substrate. The film was readily bent, even with compressive bending of the substrate, without deteriorating the structures, Figure 3d.

### 3.3. Long-Term Stability of Agar/SAC Composite Gels

One concern around gel-type electrodes is their high water content, which can produce significant volume shrinkage after drying. For practical uses, long-term sample storage is important. We investigated the stability of the agar/SAC composite gels by storing the samples in water. The cubic sample stored in air shrank within one hour, whereas the water-stored sample maintained its original shape for more than 3 months. The shrunken samples occasionally formed cracks, which is undesirable during long-term storage. The compositions of the agar10/SAC10 gels before and after water storage for more than 3 months were qualitatively compared using TGA analysis. The sample stored in water showed a slightly higher water content than the as-prepared sample. The weight ratio between the agar and SAC was found to be almost unchanged when normalized by the weight of the composite gel in the fully dried state at 200 °C, as shown in Figure 3e. These results suggest that the agar and SAC were not well-dissolved or dispersed in water for a sufficiently long time due to the high melting temperature of the agar. 

### 3.4. Conducting Property of Agar/SAC Composite Gels

#### 3.4.1. Effects of the Gel Composition, Recycling Process, and Mechanical Bending

**Figure 4:**
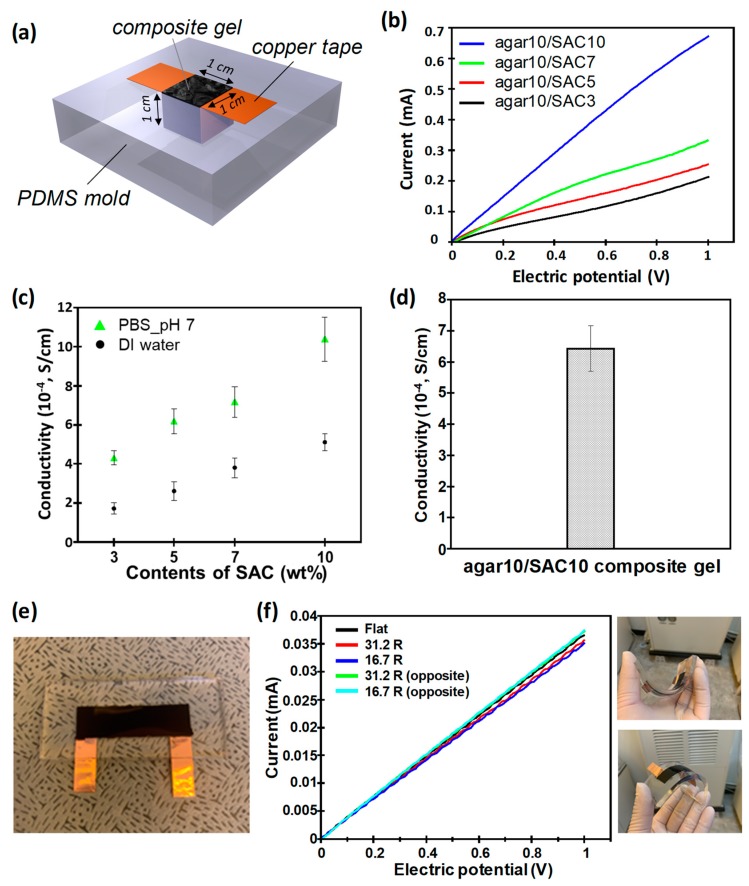
(**a**) Schematic diagram of the cubic agar/SAC composite gels in a poly(dimethylsiloxane) (PDMS) mold connected with copper taps for conducting the current–voltage (I–V) measurements. (**b**) I–V measurement of the agar/SAC composite gel prepared with various SAC contents: 10 wt % (blue), 7 wt % (green), 5 wt % (red), or 3 wt % (black) in 10 wt % agar. (**c**) The conductivity of the agar/SAC composite gel prepared with deionized (DI) water (black dot) or phosphate buffer saline (PBS) buffer (green triangle), as a function of the SAC content. (**d**) Average conductivity of recycled agar10/SAC10 composite gels. (**e**) Photo of agar/SAC composite gel integrated in flexible PDMS. (**f**) I–V measurement (left panel) and photos (right panel) of bended agar/SAC composite gel. The gel was bent in both directions with a curvature of 31.2 R (red, green) and 16.7 R (blue, cyan), respectively.

The electrical properties of the agar/SAC composite gels were investigated by varying the gel composition. We prepared identical composite gels having cube shapes of 1 cm^3^ using a PDMS mold, and they were connected to a copper tape, as illustrated in Figure 4a. Based on 10 wt % agar gel, the SAC content was varied from 3 to 10 wt % for the I–V measurements. The amount of SAC in the agar matrix was determined by considering the processability of the materials. When the amount of SAC was less than 3 wt %, the pre-gel mixture was too viscous for successful molding or extrusion, which is not preferred in broad applications. For amounts of SAC higher than 10 wt %, final agar/SAC composite gels did not have enough strength to maintain their shapes due to weak hydrogel bonding among agar polymers. The electrical properties increased as the SAC content increased, as shown in Figure 4b. The conductivity of each composite gel was calculated using the slope of the I–V curves, resulting in 1.73 × 10^−4^ S/cm for the 3 wt % SAC and 5.11 × 10^−4^ S/cm for the 10 wt % SAC composite gels. We found that the conductivity increase was not linearly proportional to the amount of SAC. Although the agar matrix has good ion transport capabilities, controlling the distance between SACs as closely as possible is crucial for the electrical properties. We think that the sample with 10 wt % SAC has enough packing density for electron transport, and the high packing density of SAC can be confirmed in Figure 2e. Such issues can be addressed by adding smaller sized conducting agents such as super p as an additive, which can fill the interstices between SACs [25].

The conductivity was further increased by replacing distilled water in the hierarchical pores of the agar matrix, with water having a higher ionic strength. We prepared the same composite gels containing phosphate buffer saline (10× PBS). The results showed that the conductivity increased by a factor of 2 for the sample prepared with distilled water, Figure 4c. It should be noted that the agar/SAC composite gel absorbed ions while maintaining its structural integrity. As long as the stability of the composite gel is guaranteed, this characteristic is advantageous for the design of electrodes that could be integrated with electrolytes while maximizing the interfacial area between the electrode and electrolyte.

The electrical properties of the agar/SAC composite gel were stable after the recycling process. To demonstrate this, we repeated the gelation and melting of the cubic agar10/SAC10 composite gel 10 times to investigate stability with the recycling process. As shown in Figure 4d, the average conductivity of recycled cubic agar/SAC composite gel was measured as 6.42 × 10^−4^ S/cm with a standard deviation of 0.73. It is noted that stable electrical properties following the recycling process are attributed to the stable sol–gel transition characteristics of the agar matrix as well as good thermal stability of the carbon-based conducting material, SAC. Although other agar-based conducting material may have similar characteristics, the significance of our demonstration is that we implanted activated carbon, which is used in industry as an electrode for commercial supercapacitors.

Maintenance of the electrical properties upon mechanical bending of the agar/SAC composite gel is crucial for soft electrodes. We integrated film-type agar/SAC composite gels in flexible PDMS as shown in Figure 4e. By considering the curvature of commercial curved screens, 4200 R, and foldable devices, 1.5 R, we measured the electrical properties of the agar/SAC composite gel by changing the curvature. As shown in Figure 4f, conductivity of agar/SAC composite gels was maintained when the curvature of the gel reached 16.7 R. There was no notable difference with bending direction. We believe that the curvature of gel can be further reduced to the curvature required by a foldable device as the thickness of agar/SAC composite gel becomes thinner.

#### 3.4.2. Recovery of Conducting Properties after Mechanical Damage of Agar/SAC Composite Gels

**Figure 5:**
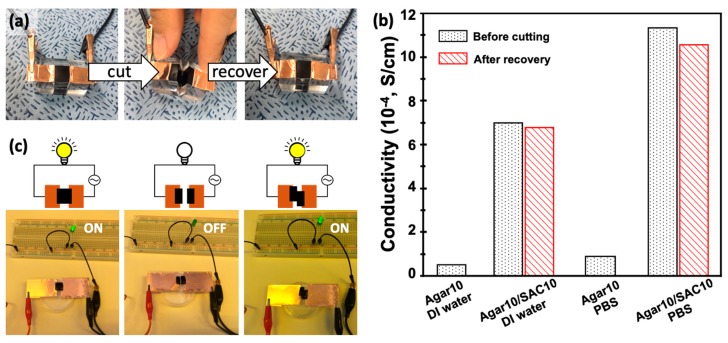
(**a**) Photos of the as-prepared (left), sliced (middle), and recovered (right) 1 cm ×1 cm ×1 cm cubic agar10/SAC10 composite gels. (**b**) Conductivity of the as-prepared (black dot) and after recovered (red stripe) agar10/SAC10 composite gels and control samples prepared with DI water or PBS buffer, respectively. (**c**) A simple electric circuit connected with the cubic agar10/SAC10 composite gels and a green light emitting diode (LED). The LED emitted green light when the circuit was connected with the agar10/SAC10 composite gel (left) whereas it turned off (middle) after slicing of the composite gel. Subsequently, the LED light turned back on as the sliced composite gels re-connected with one another, even when poorly aligned (right).

Agar/SAC electrodes are suitable for use in devices that require repeated mechanical deformation. The elastic property of the material enables repetitive stretching or bending, and the material softness enables conformal contact between the broken gels in the event that sudden mechanical damage disconnects the electrodes. The electrical properties of the agar/SAC composite gels were easily recovered due to the good deformability of the material. We measured the conductivity change of the agar/SAC composite gel before and after cutting, and compared it with that of the control samples as shown in Figure 5a. The results shown in Figure 5b revealed that the conductivity decreased by 5–7% in the recovered sample compared to the original without further treatment. Recovery from mechanical damage was further tested by preparing a simple electrical circuit system connected to a green light emitting diode (LED), Figure 5c. As the circuit was connected with the agar/SAC composite gel, the green LED turned on, whereas the light turned off when the composite gel was cut with a razor blade. Subsequently, the light turned back on as the two pieces of the agar/SAC composite gel were brought into contact with one another in a position that was slightly different from the original position. The electrical properties were readily recovered without requiring delicate alignment of the pieces of the composite gels. The facile circuit recovery was attributed to the soft and deformable characteristics of the agarose matrix, as the rough sliced gel cross-section could be readily deformed, resulting in conformal contact.

## 4. Conclusions

We developed agar/SAC composite gels for use in recyclable and shape-configurable electrodes. The temperature-responsive reversible gelling behavior of the agar/SAC mixture enabled the design of electrode shapes using a variety of processes. The highly porous SAC manifested synergetic effects on the electric properties when integrated with a porous agar matrix. The conductivity of the agar/SAC composite gels was controlled by varying the amount of SAC and the ionic strength of the aqueous medium absorbed in the agar matrix. These composite gels are potentially useful for designing energy devices with highly efficient interfaces between the electrolyte and electrode. In addition, the deformable agar/SAC composite gels permitted the ready recovery of the electrical properties of the sliced agar/SAC composite gels because the rough gel cross-section readily deformed, producing conformal contact. The agar/SAC composite gels could be further enhanced to display self-healing properties or a high stretchability, for example, by adding a self-healing material, such as poly(vinyl alcohol) [26], or by formulating the agar/SAC composite to include chemically crosslinked gels [27]. Further developments are underway and will be discussed in the near future.

## Figures and Tables

**Table 1:** Calculation of the composition of the agar and SAC in an agar/SAC composite gel based on TGA analysis.

Sample	$W_{agar+SAC}\;{}^{a}$	$W_{agar}\;{}^{b}$	$W_{SAC}\;{}^{c}$
Agar10	-	9.36%	-
Agar10/SAC5	8.83%	10.2%	6.17%
Agar10/SAC10	8.41%	10.7%	9.27%

^a^ weight percentage of the fully dried agar/SAC composite gel with respect to the swollen gel. ^b^ weight percentage of the agarose with respect to the fully dried agar/SAC composite gel. ^c^ weight percentage of SAC with respect to the fully dried agar/SAC composite gel.

## Supplementary Materials

The following are available online at https://www.mdpi.com/2073-4360/11/5/875/s1, Figure S1: Nitrogen adsorption/desorption isotherms of spherical activated carbon, Figure S2: Optical microscope images of agar/SAC composite gel with circle and line patterns using the PDMS master mold.
