# Supplementary material for: Agarose/Spherical Activated Carbon Composite Gels for Recyclable and Shape-Configurable Electrodes
Source: Polymers (Basel). 2019 May 14;11(5):875. doi: 10.3390/polym11050875 (PMC6572220; doi:10.3390/polym11050875)
Supplement: Supplementary file 1 [file polymers-11-00875-s001.pdf]

Supporting Information

Agarose/Spherical Activated Carbon Composite Gels for Recyclable and Shape Configurable Electrodes

Jong Sik Kim,[1] Ju-Hyung Kim,[1,2,*] Younghyun Cho,[3,*] and Tae Soup Shim[1,2,*]

[1]Department of Energy Systems Research and [2]Department of Chemical Engineering, Ajou University, Suwon 16499, Republic of Korea

[3]Department of Energy System Engineering, Soonchunhyang University, Asan 31583, Republic of Korea

S1. BET analysis of spherical activated carbon

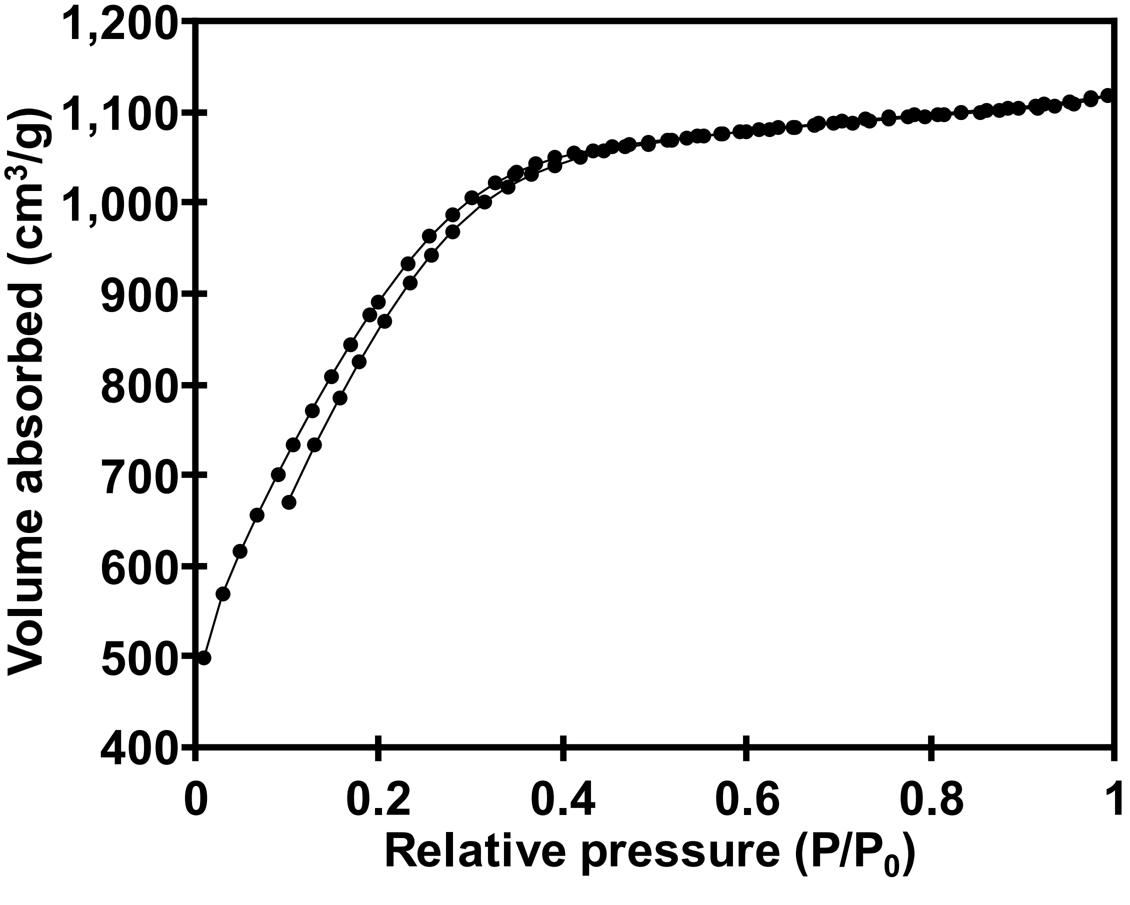

Figure S1. Nitrogen adsorption/desorption isotherms of spherical activated carbon.

S2. Experimental procedure of microstamping of agar/SAC mixture

For microstamping of agar/SAC mixture, we first prepared a mater pattern made of poly(dimethylsiloxane) (PDMS, sylgard 184, Dow Corning). Circle and line arrays were prepared by conventional softlithography. Hot molten agar/SAC at 90 °C were poured on the glass substrate and then PDMS master introduced for microstamping. Subsequently, the sample were cooled down to room temperature until agar/SAC mixture forms complete gel. The resulting agar/SAC structure, however, showed poor pattern resolution as shown in Figure S2 because of vapor evaporation during cooling process. It is attributed to the low surface moiety between PDMS and water, resulting in condensation of vapor on the PDMS surface. We addressed this problem by using hydrophilic master, poly(ethylene glycol) diacrylate (PEGDA, Mn 575, Sigma-Aldrich), which can effectively absorb water during cooling process. For hydrophilic master mold, PEGDA was mixed with 5 wt% water and 5 wt% irgacure 1173 as a photoinitiator. PEGDA mixture were infiltrated between PDMS master and glass substrate with thin film of trimethylolpropane ethoxylate triacrylate (ETPTA, Mn 428, Sigma-Aldrich). Finally, UV light (17.5 mW/cm^2) was exposed under nitrogen atmosphere to ensure complete polymerization of PEGDA. Microstamping process was done in the same manner described above and the result shows fine agar/SAC microstructures shown in Figure 3c.

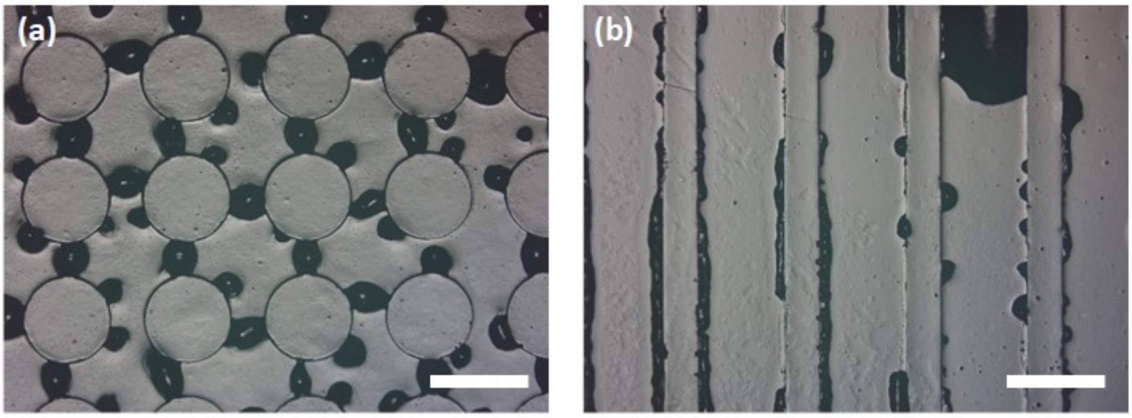

Figure S2. Optical microscope images of agar/SAC composite gel with circle (a) and line (b) patterns using the PDMS master mold. Dark area in images show vacancy in microstructure due to water condensation on PDMS mold during cooling process. Scale bars indicate 500 μm.
